# The Impact of Glycaemic Variability on Vascular Dysfunction in Diabetes

**DOI:** 10.3390/biom15111544

**Published:** 2025-11-03

**Authors:** Laura J. Offler, Liz K. Wells, Timothy M. Palmer

**Affiliations:** 1 Biomedical Institute for Multimorbidity, Hull York Medical School Centre for Biomedicine, Faculty of Health Sciences, University of Hull, Hull HU6 7RX, UK; hylo8@hyms.ac.uk; 2 School of Rehabilitation and Sport Sciences, Faculty of Health Sciences, University of Hull, Hull HU6 7RX, UK; liz.wells@hull.ac.uk

**Keywords:** glycaemic variation, endothelial dysfunction, diabetes, cardiovascular dysfunction, vascular regulation

## Abstract

It is well established that vascular dysfunction is common in people with diabetes mellitus and is associated with increased risk of heart attack, ischaemic stroke and peripheral vascular disease. Although our understanding of the molecular mechanisms responsible is incomplete, persistent hyperglycaemia observed in poorly controlled diabetes has long been thought to be a critical factor. Multiple studies have, therefore, investigated the effects of poor glycaemic control on vascular function in multiple experimental settings, from in vitro and ex vivo models of primary human cells and tissues through to pre-clinical models. This review consolidates our current understanding of how metabolic and cell signalling pathways triggered by poor glycaemic control, impact vascular function in diabetes. We also evaluate how these pathways could be exploited to develop targeted therapeutic approaches to improve cardiovascular outcomes specifically in people with diabetes.

## 1. Introduction

Diabetes mellitus (DM) constitutes a significant financial strain on global health care systems. For example, the UK spends in excess of 10 billion GBP a year on treatments and management of complications associated with DM [1]. Macrovascular complications are the leading cause of cardiovascular disease (CVD) in people with DM and conditions such as angina, myocardial infarction (MI), ischemic stroke and heart failure account for over 160,000 cases a year in the UK [2]. People with DM are also twice as likely as non-diabetic individuals to develop peripheral arterial disease (PAD), with PAD-associated diabetic foot ulcers accounting for 17% of lower limb amputations [3,4], Reduced quality of life and life expectancy is seen in patients with PAD associated foot ulcers, with patients with reporting a greater fear of lower extremity amputation than death, according to Wukich et al. [5]. According to data published by the European Society of Cardiology [6], CVD is 2–4 times more likely in patients with DM, with additional risk observed in patients with poor glycaemic control. However, several clinical trials evaluating the effects of intensive blood glucose control with diet, insulin or sulphonylurea therapy have reported little to no improvement in CVD risk, with some of these showing an increased risk of severe hypoglycaemia as a potential adverse outcome [7,8,9]. With the advent of new pharmacological treatments such as SGLT2 inhibitors, or “flozins”, and GLP-1 receptor agonists such as semaglutide, promising new studies looking into the reduction in CVD morbidity and mortality are beginning to emerge. However, the long-term risks have not yet been fully assessed, and concerns surrounding toxicity and detrimental effects have already been raised, including a cautionary link between semaglutide and pancreatitis [10].

Previously, hyperglycaemia has been proposed to be the primary cause of premature vascular dysfunction in DM, with an impairment in endothelium-dependent relaxation being the main characteristic of endothelial dysfunction (ED) [11]. While the mechanisms behind hyperglycaemia-induced ED remain unclear, focus is shifting towards investigating the impact of hypoglycaemia and glycaemic variation (GV) as potential drivers of vascular dysfunction. Several trials have now been performed in type 1 (T1DM) and type 2 (T2DM) diabetic patient cohorts to assess the relationship between GV and cardiovascular function [12,13,14,15]. While these have concluded that the incidence of both hypoglycaemia and GV posed an increased risk of developing CVD in DM, the underpinning mechanisms were not investigated, although increased oxidative stress, reactive oxygen species (ROS) production and inflammation have been proposed as possible drivers [16]. However, while there is a significant body of evidence examining the impact of hyperglycaemia, research into the effects of GV on vascular function is limited. Inflammation has been linked to oxidative stress via the activation of a pro-inflammatory transcription factor, nuclear factor kappa-light-chain-enhancer of activated B-cells (NF-kB), by superoxide radicals [17]. Previous reviews have already looked at the effect of GV on inflammatory markers such as interleukin-6, tumour necrosis factor alpha and adhesion molecules such as P-selectin, intracellular adhesion molecule-1 and vascular adhesion molecule-1 [17,18]; therefore, the focus of this review will be around vasoregulatory mechanisms.

## 2. Glycaemic Variation

**Figure 1:**
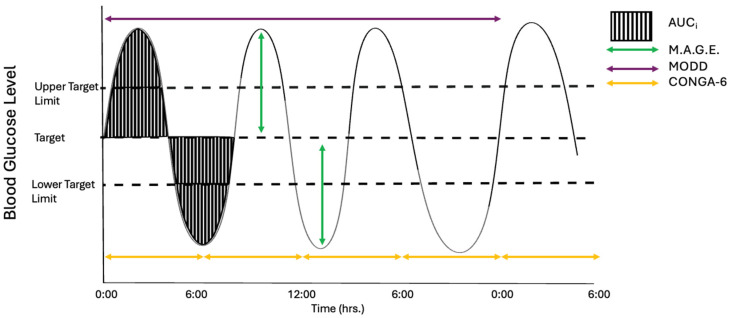
Schematic showing different measures used to assess GV: Measures include postprandial incremental area under the curve (AUC_i_), the mean amplitude of glycaemic variation (M.A.G.E), the mean of daily differences (MODD) and continuous overlapping net glycaemic action at n-hours (CONGA-n).

A search was carried out on Medline using Boolean operators to identify suitable primary research literature for review with no limitations on dates of publication (Figure S1). During initial searches, a limitation was identified around the lack of consensus in terminology for GV. Where every effort was taken to identify all relevant material for review, it is possible that other terms for glycaemic variation were not considered at the time of searching. While hyper- and hypoglycaemia are widely accepted as the correct terms to describe high and low blood glucose levels, respectively, this is not the case for GV. There is a lack of consistency in the terminology used to describe GV: glycaemic variation or variability seems to be the most common. However, phrases such as glycaemic or glucose control, glucose instability, glucose variation or intermittent hyperglycaemia seem to be used interchangeably between studies [19,20,21,22,23,24]. With each term comes marginal differences in definition and measurement, although most studies agree that variability is a fluctuation in blood glucose levels from a predefined normal range over a specific time period. There are currently over 20 markers that have been used to measure GV, some of which can be acquired from simple blood tests, such as the level of glycated haemoglobin A (HbA1c) or change in blood glucose level over time, taken via a simple finger-prick monitoring device [25]. With the adoption of continuous glucose monitoring systems (CGMs), the complexity associated with GV measurements can now be captured in people with DM in real time. Consideration of the direction of change in glucose levels, either rise or fall, amplitude of peaks and troughs, time taken for blood glucose levels to return to the healthy range, frequency of blood glucose fluctuations over time and consistency of GV patterns on a day-to-day basis all help explain the complexity of GV measurement (Figure 1) [25].

Measurement of GV can show both inter- and intra-day variability, with some measurements being more representative of change over a 24 h period while others better represent long-term changes [26]. With the development of more accurate CGM technology, it is now possible to monitor the real-time glucose status of individuals with DM to assess whether they are persistently hyperglycaemic or demonstrate an oscillating pattern of high and low blood glucose levels. The difference between these high and low peaks and the frequency the peaks occur shows the degree of variation, or glucose control, the patient experiences [27]. The mean amplitude of glycaemic excursion (MAGE), which is defined as the average of the differences between consecutive peaks and troughs of blood glucose levels that exceed one standard deviation from the mean in a 24 h period (Figure 1) and the standard deviation (SD) of blood glucose levels are two of the most commonly used intra-day variability measures [28,29]. Intra-day variation is typically observed as a rise in postprandial blood glucose levels and a subsequent fall during periods of fasting, such as overnight [25].

Studies have shown a link between increased MAGE and cardiovascular-related events in DM patients, with many showing a detrimental outcome compared to patients with either a controlled glycaemic level or a persistent hyperglycaemia, even in non-DM patients [25]. Alaska et al. [15] assessed the effect of MAGE on the number of cardiovascular events seen after percutaneous coronary intervention in patients with stable angina. The authors concluded that the cardiovascular event rate was significantly higher in patients with high MAGE compared to ones with low MAGE, irrespective of whether patients had DM [15]. Another study showed that GV affected vascular endothelial dysfunction, measuring urinary 8-isoPGF2a and flow-mediated dilation (FMD) of the brachial artery, with CGM performed over 3 days for assessment of MAGE [30]. Impaired endothelial function and urinary 8-isoPGF2a levels were both found to be increased in T2DM patients with GV, suggesting increased oxidative stress resulting from GV as a cause of vascular dysfunction.

A study by Buscemi et al. assessed GV as the percentage of the coefficient of variance (CV%) of glycaemic measurements taken on a subcutaneous continuous glucose monitor system (CSGM) every 3 min for 48 h [31]. CV% is another measure of intra-day variance. At the time of publication in 2009, the authors believed this was the first study looking at the effect of glycaemic variation on flow-mediated vasodilation as a measure of endothelial function in pre-diabetic patients. They compared CV% to MAGE and concluded that, where MAGE was designed to exclude quantitation of minor fluctuations in GV, CV% did not. This is especially significant where an alternative metabolic pathway might elicit a repair or corrective mechanism and, therefore, the true magnitude of GV might be masked when measured by MAGE alone.

The postprandial incremental area under the curve (AUC_i_) can also be determined from the CGM chart and has been used in some studies alongside MAGE to measure GV [20]. AUC is best used for measuring the degree of hyper- and hypoglycaemia following meals or periods of fasting, respectively, to allow comparison between mean AUCs (Figure 1). In one study, patients were given three meals a day, breakfast, lunch and dinner, and the mean AUCs of each group were compared over three visits [28]. Whereas MAGE measures the difference between the highest and lowest blood glucose levels, the AUC_i_ measures the duration of these changes in glucose levels. This may be clinically relevant when considering patient responses to changes in glucose levels. For example, depending on the treatment used to manage their DM, patients may have significant hyperglycaemia followed by very low blood glucose levels. This may be seen as a high MAGE but a low AUC_i_ if the rise and fall of glucose levels is steep.

One of the most widely used measurements of inter-day GV is the HbA1c level. [32]. Haemoglobin in red blood cells (RBCs) glycates when exposed to hyperglycaemic conditions. With the life span of an RBC being about 120 days, the level and duration of hyperglycaemia dictate the accumulation of glycated HbA1, allowing clinicians to assess a patient’s long-term glucose control [33]. More recently, HbA1c levels have been used to diagnose pre-diabetes as well as DM, but due to their lack of specificity and inability to reflect hypoglycaemia, it is losing favour amongst researchers as a measure of GV [34].

Recently, researchers have moved to using the continuous overlapping net glycaemic action at n-hours (CONGA-n) and the mean of daily differences (MODD) to analyse inter-day variation (Figure 1). CONGA-n is calculated by taking the blood glucose reading at time n and comparing it to the reading exactly n hours before, then calculating the SD of all readings taken. In Figure 1, n = 6, so readings are compared every 6 h, and the SD of the differences between readings would give the CONGA-6 reading. Depending on what the n value is, CONGA-n can represent either intra- or inter-day variation. The MODD is the average difference between daily blood glucose levels taken at exactly the same time on two consecutive days and is a true measure of inter-day variation [35]. Both the CONGA-n and MODD require complex calculations from CGM readings for use in the clinical setting, but the ability to produce a singular SD or mean value over a 24 h period may allow for simplification of GV modelling in the experimental setting.

## 3. Models of Investigation

Just as interrogation of the literature indicates a lack of consistency with respect to definitions of GV, it is also evident that there is variability in the protocols and models used to investigate the effects of GV on vascular function and cardiovascular risk. This is likely due to the complex arrangement of the cardiovascular system, comprising complex and specialised tissues with distinct functions [36]. For example, single-cell RNA sequencing studies have highlighted the heterogeneity of endothelial cells (ECs), smooth muscle cells (SMCs) and immune cells involved in the development of atherosclerosis [37]. Selecting the correct cell type for each model and whether to co-culture provides the first hurdle regarding consistency. Some researchers are beginning to utilise inducible pluripotent stem cells as a tractable model, but these are not without their own disadvantages, such as a lack of systemic response or large variation and heterogeneity [38]. In addition, the oxygen concentration of the blood and shear force experienced by endothelial cells vary within the cardiovascular system and are not easy to replicate in cellular models [39]. To summarise, basic in vitro cellular models do not reflect the complexity of the whole system; however, they do represent a tractable model and can therefore provide mechanistic insights for testing more complex scenarios. Several different models have already been used to look at aspects of CVD and could therefore be utilised to investigate the effects of GV going forward.

### 3.1. Animal Models

Animal models provide a more representative view of the cardiovascular system and have been used previously in the investigation of CVD treatment and management strategies [36]. Many researchers use small rodents in CVD animal models, particularly mice, which provide a tractable system with the ability to genetically validate key hypotheses in vivo. However, investigation of the literature indicates a relatively limited scope of pre-clinical in vivo studies investigating the effects of GV compared to in vitro models looking at the effects of hyperglycaemia.

There are differences between murine and human vasculature that may lead to results being non-transferable to human models. For example, wild-type mice do not naturally develop atherosclerotic lesions, so commonly used in vivo mouse models of atherosclerosis require the deletion of key genes (e.g., *Apoe*, *Ldlr*, and *Pcsk9*) that trigger hypercholesterolaemia [40]. Any results detected using murine models may, therefore, not translate to the same outcomes in human trials. The cardiovascular systems of pigs are more closely linked with humans, including heart anatomy and cellular structure [36]. Although it was possible to find several studies that looked at the effect of hyperglycaemia, once again, a study of the literature revealed few that investigated the effects of GV on vascular function in porcine models [41,42].

### 3.2. Human Models

Currently, most studies investigating the effects of GV on vascular dysfunction have been based on clinical models, correlating CGM data with specific cardiovascular outcomes [43,44,45]. One benefit of this approach is that large amounts of data can be collated due to the increased uptake of CGMs as part of DM management [43]. However, due to the chronic nature of CVD, studies can take many years to gather the data required to make an informed conclusion about CVD cause and development [46]. It is also not always easy to maintain control of longitudinal studies, which rely on strict dietary control of participants over sustained periods.

### 3.3. Cellular Models

Many cellular models have been implemented in trying to establish the molecular pathways associated with cardiovascular complications of GV. In 2002, Ido, Carling and Ruderman [47] looked at the effect of sustained hyperglycaemia on human umbilical vein endothelial cells (HUVECs) in vitro. They concluded that HUVECs needed to be cultured for a minimum of 72 h to show any apoptotic effect of supraphysiological high glucose (30 mmol/L) in their study. However, in humans, prolonged blood glucose readings of 30 mmol/L or higher are not maintained even in poorly controlled DM. A blood glucose level of 33 mmol/L is known clinically as a diabetic hyperosmolar hyperglycaemic state, suggesting there are also osmotic effects [48]. Many studies employ an osmotic control, such as mannitol, to negate any osmotic effect [49], although some studies are now suggesting hyperosmolarity alone can impact vascular function [50,51].

Ex vivo models have been used successfully to investigate glycaemic changes in the vascular system in the form of veins and arteries removed from rodent models, including rat aorta, as described by He et al. [52]. An exciting advancement in human ex vivo studies is the use of placentas for vascular studies. Already being used in other areas of research, such as neurology and foetal–maternal interactions, there is now a move to look at vascular dysfunction and thrombosis using placental vasculature as a potential flow model [53,54]. Another area in development is through the use of 3D simulated microfluidics models [55]. Such models allow the culture of multiple cell types, such as ECs and SMCs, and even platelets, under conditions that can be manipulated to represent healthy or pathological microenvironments. When investigating vascular dysfunction using such models, there are some associated challenges, such as achieving the correct shear stress rate within the system; however, if achieved, the system can be manipulated to mimic conditions such as early atherosclerosis, where it is believed that low shear stress leads to activation of pro-atherogenic factors [55]. Studies looking at the effect of hyperglycaemia on vascular dysfunction using microfluidic models have seen increased vascular dysfunction in T2DM patients compared to T1DM patients [56]; however, very few studies are looking at GV in microfluidic systems. Of the few that are, the focus leans towards reduction in vascular dysfunction in GV conditions by controlling shear stress rather than any detrimental effect of GV on the vascular system [57,58]. Microfluidic systems allow for manipulation of the microenvironment, both pharmacologically and genetically, through specifically selected modified cells and drug treatments to better understand underlying pathophysiological mechanisms. With this in mind, the lack of detailed mechanistic studies examining the effects of GV on vascular dysfunction constitutes a research gap that, if addressed with a variety of approaches, offers an opportunity to advance our understanding of the underpinning mechanisms.

## 4. Potential Mechanisms of Vascular Dysfunction

**Figure 2:**
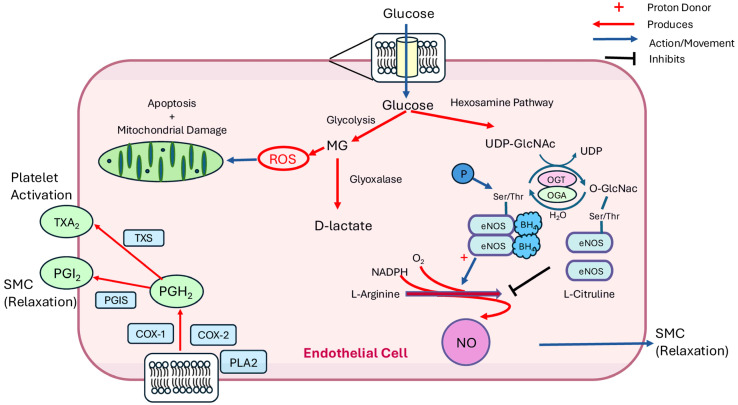
Potential effect of variable blood glucose levels on cellular apoptosis, nitric oxide (NO) production and the prostacyclin pathway in endothelial cells. Glucose entering the cell has the potential to affect several pathways. Excess methylglyoxal (MG) produced as a side product of glycolysis can increase reactive oxygen species (ROS) levels, leading to apoptosis. Uridine diphosphate *N*-acetylglucoasmine (UDP-GlcNAc), synthesised as part of the hexosamine pathway, is a substrate of *O*-GlcNAc transferase (OGT) that catalyses the transfer of *O*-linked b-N-acetylglucosamine (*O*-GlcNAc) to Ser and/or Thr residues on target proteins. A key *O*-GlcNAcylation target is endothelial NO synthase (eNOS), causing eNOS uncoupling and loss of tetrahydrobiopterin (BH_4_), which reduces NO production. *O*-GlcNAc can be removed from eNOS by the enzyme *O*-GlcNAcase (OGA). Arachidonic acid, released from cell membrane phospholipids by phospholipase A_2_ (PLA_2_), is converted to prostaglandin H_2_ (PGH_2_) by cyclooxygenases 1 and 2 (COX-1 and 2). PGH_2_ is further converted to thromboxane A_2_, which stimulates platelet activation, by thromboxane synthase (TXS) and prostaglandin I_2_ (PGI_2_), which inhibits platelet activation and triggers smooth muscle cell (SMC) relaxation by prostaglandin synthase (PGIS) [65].

In healthy haemostasis, multiple temporally co-ordinated pathways are necessary to maintain effective blood flow to prevent excessive blood loss in response to injury or infection and initiate repair. While the mechanisms behind the regulation of these pathways are complex, there are four principal elements: the vascular endothelium, platelets, coagulation factors and fibrinolytic processes [59]. The vascular endothelium controls vascular tone via interaction with underlying smooth muscle cells. When ED occurs, effective regulation of haemostasis is lost and CVD risk is increased, but again, the mechanisms behind this are not fully understood [60]. Previous studies to identify causes of hyperglycaemia-induced ED have proposed that the mechanisms lie within increased oxidative stress, increased ROS production, reduced nitric oxide (NO) production and upregulation of adhesion molecules. While the specific causes of these regulatory changes are still under investigation [61], key areas of focus have included mitochondrial dysfunction, apoptosis, proteins that regulate vascular tone and thrombosis (Figure 2), cell–cell interactions and genetic factors to better understand the increased risk of CVD among patients with DM. There are also other important mechanisms of vascular control that were not covered in this review, such as endothelin-1 and recently discovered endothelium-derived hyperpolarising factors or endothelium-derived relaxing factors, as well as neuropeptide Y released from sympathetic nerves innervating resistance vessels [62,63,64] that have been linked to vascular dysfunction but not investigated in depth alongside diabetes or glycaemic control.

### 4.1. Mitochondria and Apoptosis

As mentioned above, the ability to select gene-specific or knock-out rodent phenotypes allows researchers to narrow down areas that may be key in vascular dysfunction, although a complete understanding of the full mechanisms remains elusive. For example, a *p66^Shc−/−^* mouse model has been shown to be protected from hyperglycaemia-induced endothelial dysfunction following streptozotocin induction of T1DM [66]. Ser36 phosphorylation of the mitochondrial p66^Shc^ protein [67] is thought to increase oxidative damage and mitochondrial apoptosis [68,69]. In line with this, a study looking at the development of diabetic vascular comorbidities compared to p66^Shc^ expression in diabetic patients showed a threefold increase in vascular complications with increased baseline expression. Together, these findings and others support a role for mitochondrial apoptosis and oxidative stress in cardiovascular dysregulation (Figure 2). However, the authors were not able to find a significant correlation between hyperglycaemia and gene expression as predicted from previous studies, and concluded that the effects of hyperglycaemia may have been reduced because of the use of peripheral blood mononuclear cells instead of vascular cells.

Further studies looking at the impact of hyperglycaemia on mitochondrial function and apoptosis concluded that there are several pathways that are thought to be involved in cell death. A study by Grant et al. [70] looked at the effect of prolonged hyperglycaemia (30 mmol/L for 72 h) in HUVECs in vitro. Both caspase-3 activity, a biochemical marker of apoptosis, and mitochondrial membrane potential were measured. Caspase-3 activity was found to be increased and mitochondrial membrane potential reduced under hyperglycaemic versus normoglycaemic glucose levels (5.6 mmol/L). To identify the underlying mechanism, the authors treated the cells with 5-aminoimidazole-4-carboxamide-riboside (AICAR) to increase AMP-activated protein kinase (AMPK) activity, which reduced caspase-3 activity. They concluded that AMPK played a role in cellular protection during periods of hyperglycaemic stress. This study did not look at the effect of GV and would have benefited from further study to assess if the effect on apoptosis was increased under GV conditions [70].

However, a study by Lui et al. [71] did investigate the effect of GV, which they termed oscillating high glucose, on human coronary artery endothelial cells (HCAECs). When staining with annexin V and propidium iodide (PI) to measure apoptosis by flow cytometry, a significant increase in apoptotic cells was observed in GV-exposed cells versus those exposed to either normoglycaemic or sustained hyperglycaemic conditions [71]. Western blot analysis of the antioxidant enzyme heme oxygenase-1 (HO-1) and nuclear factor-E2-related factor-2 (Nrf2), which controls the expression of HO-1 [72], corroborated that conclusion. Nrf2 expression was reduced, leading to a reduction in HO-1 under GV conditions, which ultimately triggered increased oxidative stress. The authors concluded that the increased oxidative stress would eventually lead to increased apoptosis, although they did not directly assess whether pharmacological or genetic inhibition of either Nrf2 or HO-1 was necessary or sufficient to observe the stimulatory effect on apoptosis [71].

Further evidence supporting the hypothesis that GV increases apoptosis in ECs has been provided by a study by Risso et al. [73], in which the effects of GV versus hyperglycaemia on levels of pro-survival protein B-cell lymphoma-2 (Bcl-2) and pro-apoptosis Bcl-2-associated protein (Bax) were investigated. In HUVECs treated with variable glucose (5/20 mmol/l), levels of Bcl-2 were barely detectable after 14 days. In contrast, levels of Bax at both 7 and 14 days were significantly higher in cells cultured in GV conditions than in sustained hyperglycaemic and normoglycaemic conditions [73].

Rat aortic EC models have also been used to compare the effects of sustained hyperglycaemia versus GV on apoptosis. In a study by Wu et al., male Wistar rats were infused with a saline/ glucose mixed solution to mimic sustained hyperglycaemia (20 mmol/l constant glucose) and GV (alternating 5.5 mmol/l glucose and 20 mmol/l glucose) [74]. Markers of apoptosis, including mitochondrial membrane-localised Bax and cleaved active caspase-3, were increased, while levels of Bcl-2 and inactive pro caspase-3 were decreased in GV versus the sustained hyperglycaemic group and saline-only controls. GV also triggered increases in markers of oxidative stress and pro-inflammatory cytokine release (IL-6, TNFα) that were significantly greater than those observed in response to chronic hyperglycaemia [74].

In summary, data from multiple in vitro and in vivo approaches are consistent with a pro-apoptotic effect of GV on EC dysfunction that is associated with increased endothelial inflammation and oxidative stress. It is, therefore, clear that glycaemic control in patients with T2DM could lead to improved cardiovascular outcomes. However, further studies to better understand the underlying mechanisms are still required.

### 4.2. Oxidative Stress and Reactive Oxygen Species (ROS)

In contrast to many other cell lines, the energy requirement in ECs is considered low, with most of the adenosine triphosphate (ATP) needed produced by glycolysis [75]. It is, therefore, mitochondria that may have a prominent cell signalling role in these cells rather than simply providing energy [76]. Mitochondria are thought to be one of the biggest intracellular sources of ROS, which can be generated as byproducts of oxidative phosphorylation during ATP generation [77]. Early investigations into the effect of ROS appeared as early as 1956 when Harman wrote about the effect of ROS on ageing [78]. ROS are produced as byproducts during reduction–oxidation (REDOX) reactions and, in healthy conditions, function as signalling molecules to control key biological processes [79]. However, excessive ROS production results in cellular oxidative stress, leading to loss of function via multiple molecular mechanisms. A growing number of studies have shown that multiple lifestyle and environmental factors (e.g., poor diet and smoking) can result in excess ROS generation and oxidative stress [80]. Importantly, oxidative stress is considered a high-risk factor for DM-associated vascular complications and is thought to be one of the main contributors to vascular EC dysfunction [81].

The theory that GV in humans increases oxidative stress, potentially via increasing oxygen free radicals, has been supported by several studies evaluating oxidative stress in children and adolescents [82,83]. Elshalkami and co-authors [84] looked at the relationship between T1DM patients and oxidative stress by estimation of malondialdehyde (MDA), a product of polyunsaturated fatty acid peroxidation and a marker of oxidative stress. HbA1c levels were also assessed as a measure of inter-day variability. This study was distinct, as its focus was on GV in T1DM children, whereas other studies have focused on elderly T2DM patients, meaning the cohort being investigated had fewer confounding comorbidities. Due to the nature of CVD predominantly being a disease of later life that is not routinely seen in adolescence, it is not straightforward to assess the effect of GV on vascular dysfunction in this study. A striking increase in MDA was seen in poorly controlled T1DM patients, suggesting increased oxidative stress and potentially increased superoxide production [84]. These findings have been supported by two other studies looking at oxidative stress in T1DM children and adolescents [82,83]. Both studies not only reported an increase in MDA levels among test patients versus non-diabetic controls but also an increase in superoxide dismutase 1 (SOD-1) and a reduction in glutathione reductase (GSH) and glutathione peroxidase (GPx) levels [82,83]. SOD1, GSH and GPx are all required to convert potentially damaging superoxide radicals to molecular oxygen and water. An increase in SOD1 would be expected in response to elevated superoxide production, as it is required to catalyse the conversion of superoxide radicals to hydrogen peroxide and oxygen. However, GSH and GPx are also required to convert the generated hydrogen peroxide into water [82]. The reduction in both suggests patients with T1DM may suffer from a dysfunction in this pathway, and therefore further study would be required to assess any relationship with glycaemic control.

It has been suggested that methylglyoxyl (MG), generated either when glucose is broken down into pyruvic acid or through the degradation of glycated proteins, can increase ROS production and oxidative stress, which then increases macroangiopathy (Figure 2) [85,86,87]. MG is an electrophilic a,b-dicarbonyl aldehyde that under physiological conditions is reduced by glutathione into D-lactate. MG not only increases ROS production but can also react with protein amino groups to form advanced glycation end products (AGEs), which further accelerate ROS generation [85]. Under healthy conditions, the enzyme glyoxalase-1 inactivates MG by catalysing the isomerisation of a hemithioacetal, which is formed spontaneously from MG and glutathione, into S-D-lactoylglutathione, thereby preventing intracellular accumulation of MG. MG has been found in greater concentrations in the blood of patients with T2DM with increased GV and prolonged hyperglycaemia compared to non-diabetic patients [15]. However, a direct causal link between increased MG levels, oxidative stress and vascular dysfunction in T2DM remains to be established.

Ogawa and co-authors [85] noted that when they measured controlled spikes of post-prandial hyperglycaemia over a 3-month period and compared it to results from the previous 5 years, they found an increase in MG, which in turn led to an increase in EC dysfunction presenting as carotid intima–media thickening and vascular stiffening in T2DM patients. Although their study focused on the effects of hyperglycaemia, patients were on a modified diet and exercise regimen to induce hyperglycaemic peaks. This was assessed using HbA1c measurement over the 3 months of the study, and, in doing so, it could be considered that they were looking at the effects of GV on MG production. It is believed that MG accumulates in ECs and, therefore, even in periods of lower glucose levels, continues to cause vascular damage [85]. They concluded that MG suppressed the production of glyoxalase-1, thereby preventing MG breakdown and causing its accumulation. It has been suggested that MG reactions with arginine and lysine can form adducts such as argpyrimidine and Nε-(1-carboxyethyl)lysine (CML), respectively [88]. Argpyrimidine has been linked to atherosclerosis and is increased in atherosclerotic lesions in DM patients, while CML is a known AGE [89]. CML can form a complex with human albumin via ligand binding, which, when bound to the receptor for AGEs (RAGE) on monocytes, affects expressions of specific signalling pathways that regulate cellular viability, senescence and death, such as p38 mitogen-activated protein kinase (p38 MAPK), extracellular-signal-regulated kinase 1/2 (ERK1/2) and nuclear factor-κB (NF-κB) [88]. When considering AGEs as a cause of further oxidative stress EC dysfunction, Ogawa et al. based their conclusions on previous research by Chen et al., who used Dahl salt-sensitive rats [85,90]. In this study, rats showed increases in AGEs and RAGE activity with decreased renal function when MG levels were increased. Oxidative stress from RAGE/AGE interactions has also been shown to activate NF-kB and cell adhesion molecules in ECs that lead to vascular dysfunction.

Maeda et al. [23] looked further at the link between GV and the role of superoxides by examining the effect of post-prandial hyperglycaemia on cellular senescence in HUVECs. It was hypothesised that the reduction in vascular function was due to increased cellular senescence caused by increased ROS production when cells were exposed to extreme post-prandial hyperglycaemic changes. The study specifically looked for intracellular ROS, hydrogen peroxide, hydroxyl radicals and peroxynitrite from HUVECs cultured under normoglycaemic (5.6 mmol/L), hyperglycaemic (20 mmol/L) and GV (alternating 5.6/20 mmol/L) glucose conditions [23]. Increased senescence was reported in the constant hyperglycaemic cells and, more significantly, in the cells exposed to the GV regimen. A significant increase in superoxide anion levels was seen in the GV conditions compared to constant hyperglycaemic and normoglycaemic conditions, suggesting GV specifically affects superoxide production [23]. This led the authors to investigate the nicotinamide adenine dinucleotide phosphate (NAD/NADPH) oxidase system, which generates superoxide anion by transferring electrons from NADPH to molecular oxygen [91]. Interestingly, the authors found an upregulation of the CYBA gene expression in GV but not in hyperglycaemic conditions. As CYBA encodes p22^phox^ [92], a critical membrane-bound subunit of the NADPH oxidase complex, the authors concluded that NADPH oxidase-derived superoxide anions played a significant role in EC senescence in response to GV, but further study is needed to pinpoint the mechanism in detail, as they could not specify which NAPDH oxidase (NOX) isoforms were responsible for the senescence changes [93]. Of the seven NOXs, NOX-1,2, 4 and 5 are the predominant isoforms found in vascular cells, with all requiring activation by a p22^phox^ subunit and NOX-1, 2 and 5 being known to produce superoxide anions and NOX-4 and 5 producing H_2_O_2_ [94]_._ In other studies using a *db/db* T2DM mouse model, reduced endothelial-dependent relaxation responses and Ser1177 phosphorylation of eNOS in mesenteric resistance arteries isolated from T2DM mice were observed alongside increased p22^phox^ expression and increases in p38 MAPK and ERK1/2 phosphorylation and activation [95]. Importantly, these changes in vasorelaxation and protein phosphorylation could be reversed either by siRNA-mediated in vivo depletion of p22^phox^ or infusion of PEGylated SOD [73]. In summary, these findings suggested that in T2DM mice, p22^phox^-mediated generation of ROS triggered the activation of p38 MAPK and ERK1/2 while also reducing Ser1177 eNOS phosphorylation to reduce endothelial-dependent vasorelaxation.

### 4.3. Arginase and Nitric Oxide

Nitric Oxide (NO) has two main critical functions that impact the vasculature to control the supply and demand of oxygen: the first is the regulation of vascular tone via NO diffusion from the endothelium to activate soluble guanylate cyclase in underlying vascular smooth muscle, triggering a signalling cascade that results in vasodilatation. NO also controls mitochondrial oxygen consumption by inhibiting cytochrome *c* oxidase [96]. In addition, NO also exerts anti-inflammatory and anti-thrombotic effects [97]. NO is predominantly produced by nitric oxide synthases (NOSs) of which there are three isoforms, with eNOS (NOS3) being the primary isoform in ECs responsible for the production of NO from L-arginine in the presence of molecular oxygen and NADPH (Figure 2) [98]. L-Arginine can also be converted to ornithine and urea by the metalloenzyme arginase [99]. It has been estimated that approximately 1% of the daily intake of L-arginine is metabolised to NO by eNOS with citrulline as a byproduct [100]. However, as they use the same substrate, arginase can compete with eNOS, providing an important mechanism by which NO production can be controlled, and may also trigger a so-called “uncoupling” of eNOS in which it switches from NO production to superoxide production [101].

A study by Mahdi et al. [100] looked at the effect of intense glucose-lowering therapy on endothelium-dependent vasodilatation (EDV) and endothelium-independent vasodilatation (EIDV) when using arginase inhibitors. Blood samples were taken from T2DM patients to measure arginase activity via plasma measurement of its substrate L-arginine and its product L-ornithine, with vascular function assessed by forearm blood flow using venous occlusion plethysmography [100]. They concluded that there was an improvement in EDV but not in EIDV when arginase was inhibited prior to glucose-lowering treatment. However, after an intensive 8-week glucose-lowering treatment regimen consisting of a diet, lifestyle education and continuous glucose monitoring, there was no increase in the effect of EDV after arginase inhibition, but there was a notable increase in EIDV. The authors believe there are two mechanisms that cause vascular EC dysfunction, long-term, in DM patients [100]. One mechanism they proposed was that promoter regions in genes encoding eNOS and also those involved in ROS formation, such as NOX4, undergo irreversible epigenetic modifications responsible for the so-called “metabolic memory”. Evidence for this has been supported in a study by Liao et al. [81], who looked at H3K3 and H3K9 methylation in the promoter regions of NOX4 and eNOS genes, and found changes in methylation consistent with reported markers of metabolic memory in NF-kB target genes [102,103]. A second theory proposed was that AGE accumulation triggered by hyperglycaemia and oxidative stress in T2DM promotes activation of RAGEs, which induces EC inflammation and subsequent vascular remodelling, leading to blood vessel narrowing [104]. A potential role of arginase in both these models is supported by the ability of arginase inhibitors to reduce ROS and increase NO production, thereby reducing oxidative stress [100].

Early studies on human aortic ECs demonstrated that prolonged exposure to hyperglycaemia (22.2 mmol/L) increased eNOS mRNA and protein levels compared to a normoglycaemic 5.5 mmol/L exposure. These were accompanied by increased levels of nitrite, which is a surrogate marker of NO production, in hyperglycaemic cells [11,102]. In vivo studies have also assessed forearm blood flow after brachial artery infusion in response to *N*^G^-monomethyl-l-arginine (L-NMMA, which binds and competitively inhibits eNOS), methacholine chloride (MCh, a muscarinic acetylcholine receptor agonist that activates eNOS in ECs) and sodium nitroprusside (a NO donor that bypasses eNOS and was used to define endothelium-independent NO-mediated vasodilation) in well-controlled versus poorly controlled T1DM patients in which cholesterol and insulin levels were matched [105]. While they found vascular responses to each of L-NMMA, MCh and SNP were reduced in poorly controlled T1DM patients; these were all significantly improved by a relatively short (3-week) period of improved glycaemic control [84]. Interestingly, infusion of bovine copper–zinc SOD was able to specifically rescue responsiveness to MCh in the poorly controlled group, indicating a role for ROS in compromising receptor-mediated eNOS activation [105]. It is worth noting that this study was performed in patients diagnosed with T1DM in the previous 3 years without any previous clinical indication of vascular dysfunction, whereas many previous studies have involved patient cohorts with longstanding DM and pre-existing vascular complications [105]. This suggests that early effective interventions could reduce the risk of CVD in DM patients.

To summarise, where studies evaluating the contribution of the NO pathway under GV conditions are scarce, the evidence supporting significant effects of sustained hyperglycaemia characteristic or poor glycaemic control effects once again presents multiple areas of interest for further research. As with studies examining mitochondrial involvement and oxidative stress, these should focus on ROS involvement and their impact on post-translational modifications of target proteins, such as histone methylation, involved in sustained vascular dysfunction in DM.

### 4.4. O-GlcNAcylation

In the late 1990s, studies started to look at post-translational modification (PTM) by *O*-linked N-acetylglucosamine (*O*-GlcNAc) on serine and threonine residues and its association with increased CVD risk [106]. Protein *O*-GlcNAcylation has been shown to be increased in vascular ECs under hyperglycaemic conditions, with several mechanisms previously reported to be involved in increasing the risk of CVD, as concluded by Bolanle et al. [106]. One such mechanism is the occupation of regulatory Ser and Thr phosphorylation sites on target proteins such as eNOS [107]. It is possible that this process reduces the availability of active Ser1177-phosporylated eNOS, and potentially causes eNOS uncoupling, leading to vascular dysfunction (Figure 2). Evidence supporting this hypothesis has been reported by Du et al. [108], investigating the effects of hyperglycaemia on eNOS activity and Ser1177 phosphorylation catalysed by protein kinase B (AKT) [109]. Du et al. have proposed that overproduction of mitochondrial superoxide in response to hyperglycaemia is the prime mechanism causing vascular dysfunction, especially when linked with the hexosamine pathway [110,111]. Approximately 3–5% of the cell’s glucose intake is converted into uridine-diphosphate-N-acetylglucosamine (UDP-GlcNAc), which is required for glycosylation, where the *O*-GlcNAc moiety is transferred to the target protein [106]. They demonstrated that hyperglycaemia increased UDP-GlcNAc levels in bovine aortic endothelial cells (BAECs) and inhibited eNOS activity by blocking phosphorylation at Ser1177 [108]. Interestingly, they also found that GlcNAc was able to modify other eNOS residues, but these were not upregulated in hyperglycaemic conditions. This finding was in agreement with data produced by Beleznai and Bagi [112], who looked at arteriolar dilatation in rat gracilis muscles under hyperglycaemic conditions. They found an increase in protein *O*-GlcNAcylation in femoral arteries exposed to high glucose (30 mM) and, although there was no detectable increase in eNOS protein, found reduced Ser1177 phosphorylation of eNOS in hyperglycaemic conditions [112].

While both the above studies looked at hyperglycaemia, He et al. [52] investigated the effect of glucose deprivation on *O*-GlcNAcylation of eNOS. Interestingly, an increase in eNOS *O*-GlcNAcylation was associated with a decrease in Ser1177 phosphorylation when BAEC and Sprague-Dawley rat aortas were exposed to decreasing glucose concentrations. However, in contrast to the hyperglycaemia studies, this led to an increase in NO production and was associated with increased *O*-GlcNAcylation of Thr866 [52]. To the best of our knowledge, no studies have examined protein *O*-GlcNAcylation under variable glucose conditions. Any such study would hopefully provide further insight into the ability of eNOS to perform multiple modifications, including any switching between *O*-GlcNAcylation at Ser1177 and Thr866, to regulate NO production in response to GV.

## 5. Conclusions

Although it appears that extensive research has been performed investigating the effects of blood glucose levels and the increased risk of CVD in patients with DM, most studies have focused on the effects of sustained hyperglycaemia. As discussed earlier in this review, some have inadvertently looked at GV in the form of postprandial hyperglycaemic change. However, very few have considered the effect that increased glucose variability, including the fall to extreme hypoglycaemic conditions, could have. This may be in part due to the lack of a consensus in defining glycaemic variability, which, if standardised, would allow researchers to better consolidate knowledge. This would also be facilitated by an agreed approach to assessing GV in patients, where there is currently none. In addition, while there has been some progress in the effect of GV on CVD when looking at the NO pathway, it is clear that further study is required in all other potential mechanisms discussed, including apoptosis and *O*-GlcNAcylation. One area where it was not possible to find any studies looking specifically at the effect of GV on CVD was with respect to the prostaglandin pathway (Figure 2). Given that it is vital in the control of SMC relaxation and platelet activation, this would be a productive avenue for future investigation.

## Data Availability

No new data were created or analyzed in this study.

## Supplementary Materials

The following supporting information can be downloaded at: https://www.mdpi.com/article/10.3390/biom15111544/s1, Figure S1: Literature flow chart.

## Abbreviations

The following abbreviations are used in this manuscript:

8-ISOPGF2α	8-Isoprostaglandin F2α
AGE	Advanced glycation end product
AICAR	5-Aminoimidazole-4-carboxamide ribonucleotide
AKT	Protein kinase B
AMP	Adenosine monophosphate
AMPK	AMP-activated protein kinase
ATP	Adenosine triphosphate
AUCi	Incremental area under the curve
BAECs	Bovine aortic endothelial cells
BAX	Bcl-2-associated protein
Bcl-2	B-cell Lymphoma 2
CGM	Continuous glucose monitor
CONGA-n	Continuous overlapping net glycaemic action at n-hours
CSGM	Subcutaneous CGM
CV%	Coefficient of variance
CVD	Cardiovascular disease
DM	Diabetes mellitus
DNA	Deoxyribonucleic acid
EC	Endothelial cell
ED	Endothelial dysfunction
EDV	endothelium-dependent vasodilatation
EIDV	endothelium-independent vasodilatation
eNOS	Endothelial nitric oxide synthase
FMD	Flow-mediated dilatation
GLP1	Glucagon-like peptide-1
GV	Glycaemic variation
HbA1c	Glycosylated haemoglobin A1
HUVEC	Human umbilical vein endothelial cell
L-NMMA	NG-Monomethyl-L-arginine
MAGE	Mean amplitude of glycaemic excursion
MCH	Methacholine chloride
MDA	Malondialdehyde
MG	Methylglyoxyl
MI	Myocardial infarction
MODD	Mean of daily differences
NAD	Nicotinamide adenine dinucleotide
NADPH	NAD phosphate
NO	Nitric oxide
NOS	Nitric oxide synthase
O-GlcNAc	O-linked β-N-acetylglucosamine
PAD	Peripheral arterial disease
PKC	Protein kinase C
PTM	Post-translation modification
RAGEs	AGE receptors
RBC	Red blood cell
REDOX	Reduction–oxidation
ROS	Reactive oxygen species
SGLT-2	Sodium–glucose cotransporter-2
SD	Standard deviation
SMC	Smooth muscle cell
SNP	Sodium nitroprusside
T1DM	Type 1 diabetes mellitus
T2DM	Type 2 diabetes mellitus
UDP-GlcNAc	Uridine-diphosphate-GlcNAc
