# Supplementary material for: The Impact of Glycaemic Variability on Vascular Dysfunction in Diabetes
Source: Biomolecules. 2025 Nov 3;15(11):1544. doi: 10.3390/biom15111544 (PMC12650407; doi:10.3390/biom15111544)
Supplement: Supplementary file 1 [file biomolecules-15-01544-s001.zip › biomolecules-3921462-supplementary.pdf]

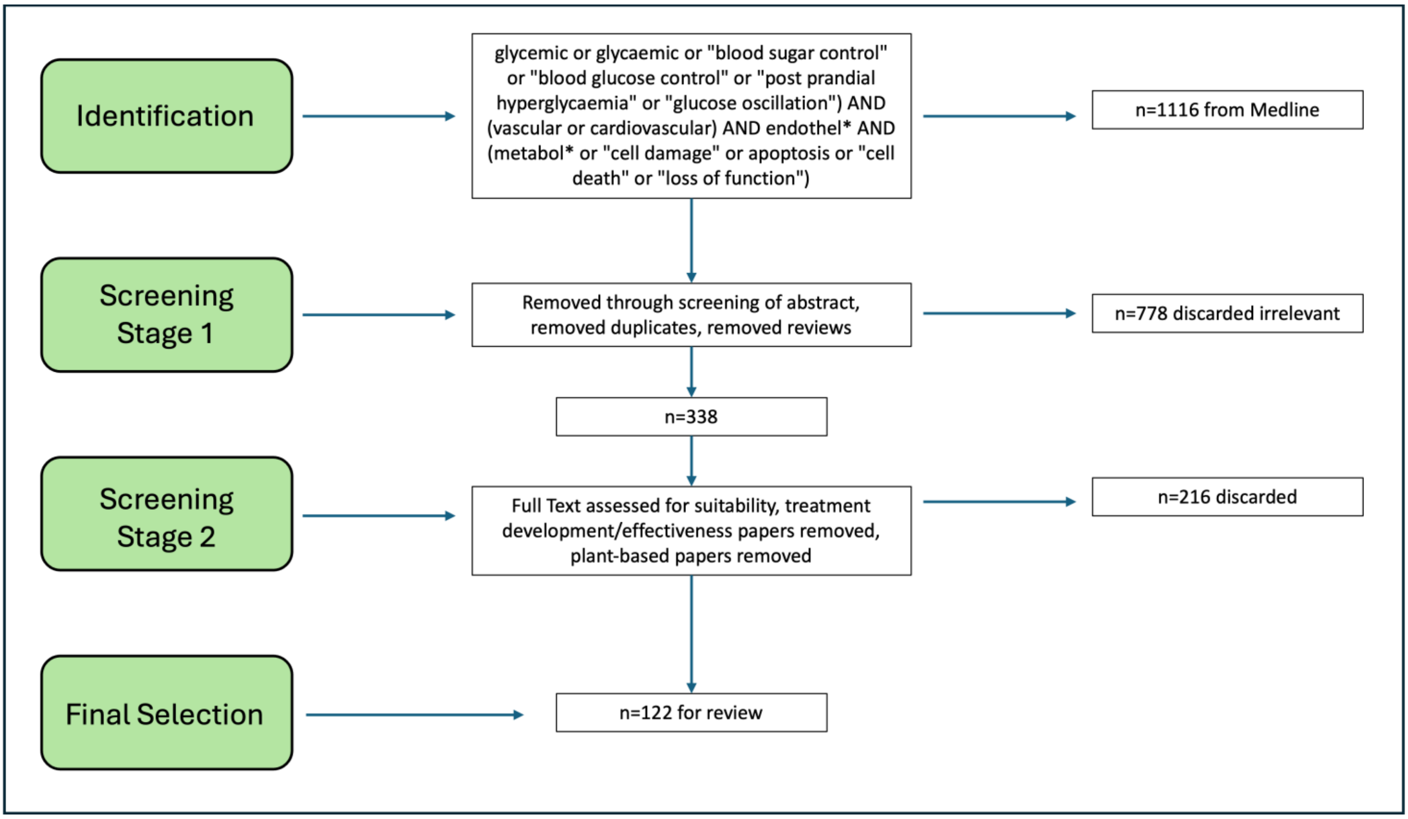

Figure S1: Literature flow chart showing the number of records identified from data searching, how many were excluded, and the final number of studies included in the review.

.
